# A retrospective observational study of 1000 consecutive patients tested with the FilmArray® Meningitis/Encephalitis panel: clinical diagnosis at discharge and microbiological findings

**DOI:** 10.1038/s41598-024-54621-9

**Published:** 2024-02-18

**Authors:** Torgny Sunnerhagen, Johan Widén, Sahar Handhal, Gülşen Özkaya Şahin

**Affiliations:** 1 Clinical Microbiology, Infection Prevention and Control, Office for Medical Services, Region Skåne, Lund, Sweden; 2 Division of Infection Medicine, Department of Clinical Sciences Lund, Lund University, Lund, Sweden; 3 Clinic of Infectious Diseases, Skåne University Hospital, Lund, Sweden; 4 Division of Medical Microbiology, Department of Laboratory Medicine Lund, Medical Faculty, Lund University, Lund, Sweden

**Keywords:** Outcomes research, Infectious diseases, Laboratory techniques and procedures, Clinical microbiology

## Abstract

FilmArray® Meningitis/Encephalitis panel (FAME-p) is used to diagnose central nervous system (CNS) infections. In this study, we investigated performance of FAME-p compared to comparator assays (CA), and for the first time, clinical diagnosis at discharge (CDD). 1000 consecutive patients with a cerebrospinal fluid (CSF) sample analyzed with FAME-p were identified. As CA, culture, polymerase chain reaction and cryptococcal antigen test were used. Medical records of patients were obtained. A CDD of CNS infection was made in 139 of 1000 CSF samples. FAME-p was positive in 66 samples with 44 viral and 22 bacterial agents. Thirteen FAME-p findings were not confirmed by CA, with four discrepant results remaining after comparison with the CDD. Positive percentage agreement (PPA) calculated against CA was 100%. Negative percentage agreement (NPA) calculated against CA was 94.4–99.8% for *Haemophilus influenzae*, *Listeria monocytogenes*, *Streptococcus agalactiae*, *S. pneumoniae* and varicella-zoster virus (VZV). NPA calculated against CDD was higher (compared to CA) for *L. monocytogenes*, *S. agalactiae* and VZV (100%), and lower for *Escherichia coli*, enterovirus and herpes simplex virus 2 (50–83.3%). NPA of FAME-p for human herpes virus 6 was difficult to interpret. Eighty-four cases received diagnosis of CNS-infection despite negative FAME-p. The four most common non-infectious etiologies were primary headache disorders, cranial nerve palsies, neuroinflammatory disorders and seizure. Although FAME-p shows good performance in diagnosis of CNS infections, result of FAME-p should be interpreted carefully. Considering infectious diseases not covered by FAME-p as well as non-infectious differential diagnoses is important in this context.

## Introduction

Infections in the central nervous system (CNS) are life-threatening and may generate sequelae. Early diagnosis is complicated owing to the wide spectrum of microorganisms that can be involved, time-consuming microbiological diagnostic methods as well as the broad range of other diseases that could mimic CNS infections, i.e. autoimmune encephalitis, CNS-tumor, cerebrovascular disorders and migraine^1,2^.

The FilmArray® Meningitis/Encephalitis (FAME-p) (Bio-Fire Diagnostics, Biomérieux Company, UT, USA) is a molecular diagnostic test using an automated multiplex polymerase chain reaction (PCR) system that simultaneously identifies 14 pathogens including *Escherichia coli* K1*, Haemophilus influenzae, Listeria monocytogenes, Neisseria meningitidis, Streptococcus pneumoniae*, cytomegalovirus (CMV), enterovirus (EV), herpes simplex virus 1 (HSV-1), HSV-2, human herpesvirus 6 (HHV-6), human parechovirus (HPeV), varicella-zoster virus (VZV) and *Cryptococcus neoformans/Cryptococcus gattii* in cerebrospinal fluid (CSF). At this point, FAME-p has been implemented in routine laboratory diagnostics for CNS infections and provides fast results and limited laboratory hands on work.

Although the performance of this system has been evaluated in detail in several studies, agreement on advantages and disadvantages of FAME-p is still controversial. This could be explained by one-sided discrepancy analysis of FAME-p, comparison only with routine microbiological diagnostic procedures and not always accounting for clinical diagnosis at discharge (CDD)^3–12^.

This retrospective observational study of 1000 patients represents, to the best of our knowledge, the first study to run a two-sided discrepancy analysis of FAME-p compared to routine microbiological tests and CDD. This study aims to contribute to fill knowledge gap concerning performance and interpretation of FAME-p.

## Materials and methods

FAME-p (CE-marked and FDA-approved) was implanted in clinical practice in March 2020 at the Clinical Microbiology Laboratory in Lund, Sweden, and was available to clinicians in the region. This laboratory serves all public and private in-patient and out-patient clinics in Skåne County with total population of 1.4 million. For this observational study, CSF samples referred for diagnosis of CNS infection were included. CSF was obtained though lumbar puncture. Transportation from the hospitals to the clinical microbiology laboratory was done several times per day, with samples stored in a fridge if analysis was not started immediately. The inclusion period was from April 2020 to August 2021, during which FAME-p was done in the clinical microbiology laboratory during office hours Monday to Friday, and during Saturdays. Exclusion criteria were inaccessible electronic medical records and CSF taken at a clinic out of Skåne County. Repeat specimens from the same patient were excluded and only the first specimen and disease episode were used for analysis in the study.

The medical records of all patients were reviewed for demographic profiles, CSF biochemistry results [white blood cell count (WBC) and differential, CSF/blood glucose ratio, protein, and albumin], and CDD.

As a principle for observational study, microbiological analyses were performed according to the decisions made by the clinical microbiologist on duty in collaboration with the physician in charge of patient care, according to standard principles using bacterial/fungal culture, 16S ribosomal (r) DNA PCR, in-house PCR and cryptococcal antigen lateral flow assay (CALFA, Immy, Norman, OK) as appropriate. Due to the retrospective nature of the study, the investigators did not influence the choice of analyses performed nor the diagnosis registered. The datasets analyzed during the current study are available from the corresponding author on reasonable request.

### FAME-p testing

This study was conducted using FilmArray® Torch instrument and FilmArray® ME Panel CE IVD 6 30 Pack kit, and analysis was performed according to the manufacturer’s instructions (4).

### Comparator testings

As bacterial comparator testing, bacterial culture, 16S rDNA PCR and in-house PCR were used. Bacterial cultures were performed using direct plating samples on blood agar incubated in CO_2_ enriched atmosphere, and fastidious aerobe agar incubated under anaerobic conditions, as well as inoculating a part of the sample in tryptic soy broth and brain–heart incubated in CO_2_-enriched atmosphere. All incubations were done at 37 °C, with the samples being incubated for a total of 7 days as standard. Dedicated fungal culture was not routinely done except at the request of the clinic sending the cerebrospinal fluid, though extra plates such as Saboraud agar could be added at the discretion of the laboratory technician or clinical microbiologist in charge of sample analysis. For identification of bacterial colonies, matrix-assisted laser desorption ionization-time of flight mass spectrometry was used (MALDI-TOF MS, Bruker Daltonics, using the Bruker MBT Compass library version most recent at the time of sample analysis).

The protocol for 16S-rDNA PCR used primers TGCCAGCMG CCGCGGTWAT as forward primer and ACCATYTCACRACACGAGCT as reverse primer, with the amplified fragment approximately 570 bp as described in previous publications^13,14^.

For identification of *H. influenzae, N. meningitidis* and *S. pneumoniae,* an in-house PCR was used. The in-house PCR is directed against *CtrA* for *N. meningitidis*, against *glpQ* for *H. influenzae* and *lytA* for *S. pneumoniae*. As viral comparator testing, in-house PCR was used to identify HSV-1 (forward primer AGGAGGGGTATAACAAAGTCTGTC, reverse primer ATAACTGATGATCGGGGTAGTTGGTC), HSV-2 (forward primer CCCATCCTCCTTCGGCAGTA, reverse primer GCCGCCCTGGTACGTGTA), VZV (forward primer TTGACGGCCAATTGTAGTGACA, reverse primer CGGAAGTTCTTCAGATGAAGCAGTG), CMV (forward primer TCGCGCCCGAAGAGG, reverse primer CGGCCGGATTGTGGATT), EV (forward primer GGTGCGAAGAGTCTATTGAGC, reverse primer CACCCAAAGTAGTCGGTTCC) and HPeV (forward primer CTGGGGCCAAAAGCCA, reverse primer GGTACCTTCTGGGCATCCTTC). For HHV-6, comparator testing with in-house PCR was performed at Sahlgrenska University Hospital, Department of Clinical Microbiology, Gothenburg, Sweden. As cryptococcal comparator testing, a CrAg lateral flow test from Immy (CALFA) was used.

### Discrepancy analysis

In the initial analysis, FAME-p result was considered true positive (TP) or true negative (TN) when it was confirmed by comparator method. Discrepant results were considered false positive (FP) if FAME-p could not be confirmed using comparator method, and false negative (FN) if the agents were identified using comparator method but not by FAME-p. Discrepancy analysis was further examined through CDD. Since CDD was collected for all FAME-p specimens, positive and negative predictive values (PPV and NPV, respectively) were also calculated.

### Statistical analysis

Statistical analyses were performed using IBM SPSS 27 and MedCalc. Positive percentage agreement (PPA) was calculated as 100 × [TP/(TP + FN)], and negative percentage agreement (NPA) as 100 × [TN/(TN + FP)]. For comparison between FAME-p positive and negative results, the chi-square or Fisher’s exact test for the analysis of categorical variables and Mann–Whitney’s two-sample test for continuous variables were used. Kruskal–Wallis test was used to compare age groups. An odds ratio (OR) with 95% confidence interval (CI) was used to test differences within groups.

### Ethical considerations

The study was approved by the Swedish Ethical Review Authority (Stockholm, Department 4 Medicine, 2021-04407). Due to the observational design, the need for informed consent was waived by Swedish Ethical Review Authority (Stockholm, Department 4 Medicine, 2021-04407). The research was performed in accordance to the declaration of Helsinki.

## Results

### Patient cohort

Using the inclusion and exclusion criteria defined above, 1172 consecutive samples were retrospectively identified in the study. After excluding 71 samples sent from laboratories in other Swedish regions, and 101 duplicate samples, 1000 samples from the same number of patients were included for further analyses. Transportation from the hospitals to the clinical microbiology laboratory was done several times per day, with the median time from lumbar puncture to registration at the start of sample analysis was 11.5 h.

### Patient characteristics and clinical diagnoses

In a primary analysis, patients were categorized into those with findings in FAME-p and those with a negative FAME-p. Reviewing the medical records, patients were classified as either having a CNS infection or not, according to CDD. Demographic data as well as biochemical analyses (cell counts, lactate, albumin and protein analyses, lactate, and glucose) from CSF are presented in Table 1. Patients with a positive FAME-p were significantly more likely to have a clinical diagnosis of CNS infection compared to patients with a negative FAME-p (83.3% and 8.7%, respectively; p < 0.001). In accordance with this result, patients with positive FAME-p, compared to those with negative FAME-p, had significantly higher amounts of WBC, albumin, total protein and lactate, as well as lower glucose levels and CSF/blood glucose ratio in CSF. The proportion of patients with abnormal CSF values were significantly higher in patients with a positive FAME-p for analytes where normal ranges were applicable: glucose ratio, albumin, and WBC (p < 0.001 for all). Of the 1000 patients included in the study, 661 had normal CSF white blood cell counts. No significant differences in age or gender were seen between the groups.

**Table 1:** Demographics of patients and biochemical analysis of CSF.

	FAME-p positive, total (n = 66)	FAME-p negative (n = 934)	p value^1^
Median age (year) (IQR)	37.0 (12.5–65.2)	48.0 (21.8–68.0)	0.18
Gender (% women)	40.9	47.0	0.37
Diagnosis, CNS infection (%)	83.3	8.7	< 0.001
CSF analysis			
Median PMNL (cells/µL) (IQR) [n]	< 3 (< 3 to < 3) [68]	< 3 (< 3 to < 3) [917]	< 0.001
Median MNL (cells/µL) (IQR) [n]	38 (13.5–66) [68]	< 3 (< 3 to < 3) [917]	< 0.001
Median protein (mg/L) (IQR) [n]	0.75 (0.41–1.0) [30]	0.37 (0.25–0.56) [475]	< 0.001
Median lactate (mmol/L) (IQR) [n]	2.7 (1.9–4.8) [65]	1.9 (1.6–2.5) [857]	< 0.001
Median albumin (mmol/L) (IQR) [n]	496 (261–1277) [60]	220 (147–351) [857]	< 0.001
Median glucose (mg/L) (IQR) [n]	3.6 (2.8–4.3) [65]	4.0 (3.6–4.7) [858]	< 0.001
Median CSF/blood glucose ratio (IQR) [n]	0.58 (0.39–0.63) [54]	0.66 (0.57–0.73) [636]	< 0.001

*CSF* cerebrospinal fluid, *FAME-p* FilmArray® Meningitis/Encephalitis panel, *IQR* interquartile range, *CNS* central nervous system, *PMNL* polymorphonuclear leucocytes, *MNL* mononuclear leucocytes.

^1^p values calculated using the Mann–Whitney’s two-sample test, comparing means over the columns, the chi-square test, or Fischer`s exact test when appropriate.

### Performance of FAME-p

FAME-p was positive in 66 cases (22 cases with bacterial agents and 44 cases with viral agents) and negative in the remaining 934 cases (Table 2). The most prevalent bacterial agents were *S. agalactiae* (n = 9) and *S. pneumoniae* (n = 6). The highest detection rates for bacterial agents were in children (n = 12). Concerning viral agents, the highest detection rates were in adults (18–64 years, n = 24) and VZV was the dominating agent (n = 23). No cases of multiple pathogens being identified by FAME-p in the same sample occurred.

**Table 2:** Total number of FilmArray® Meningitis/Encephalitis panel (FAME-p) analyte detections by total positive samples detected and age group.

Analyte	FAME-p result				
	No. detected	% of positive samples	No. of positive detections by age group		
			< 18 year	18–64 year	≥ 65 year
Bacteria					
*Escherichia coli* K1	1	1.5	1	0	0
*Haemophilus influenzae*	1	1.5	0	1	0
*Listeria monocytogenes*	3	4.5	0	1	2
*Neisseria meningitidis*	2	3	1	1	0
*Streptococcus agalactiae*	9	13.7	7	0	2
*Streptococcus pneumoniae*	6	9.1	3	0	3
Total	22	33.3	12	3	7
Viruses					
Cytomegalovirus	1	1.5	0	0	1
Enterovirus	2	3	1	1	0
Herpes simplex virus 1	3	4.5	1	1	1
Herpes simplex virus 2	5	7.6	0	4	1
Human herpes virus 6	10	15.2	3	6	1
Human parechovirus	0	0	0	0	0
Varicella-zoster virus	23	34.8	3	12	8
Total	44	66.6	8	24	12
Yeast					
*Cryptococcus neoformans/C. gattii*	0	0	0	0	0

No cases of pathogens being missed by FAME-p but being detected by CSF culture, PCR or CALFA occurred, but 18 cases of positive FAME-p occurred with the respective microbiological tests being negative (Table 3). The PPA of FAME-p was 100% for 11 of 14 analytes. Three analytes, *H. influenzae*, HPeV and *C. neoformans/C. gattii*, were neither detected by FAME-p nor comparatory assays, and therefore PPA could not be calculated. FAME-p demonstrated a NPA of 100% for 8 of 14 analytes: *E. coli* K1, *N. meningitidis*, CMV, EV, HSV-1, HSV-2, HPeV and *C. neoformans/C. gattii*. Six analytes had lower specificities: 94.4–99.8% for *H. influenzae, L. monocytogenes, S. agalactiae, S. pneumoniae,* and VZV, and 50% for HHV-6. Some pathogens were only detected in a few cases, influencing the confidence intervals of both PPA and NPA (Table 3).

**Table 3:** Performance analysis of FilmArray® Meningitis/Encephalitis panel (FAME-p) vs comparatory assays and clinical diagnosis at discharge (CDD).

Analyte	FAME-p vs comparatory assays				FAME-p vs clinical diagnosis at discharge							
	Positive percentage agreement		Negative percentage agreement		Positive percentage agreement		Negative percentage agreement		Positive predictive value		Negative predictive value	
	TP/(TP + FN)	% (95% CI)	TN/(TN + FP)	% (95% CI)	TP/(TP + FN)	% (95% CI)	TN/(TN + FP)	% (95% CI)	TP/(TP + FP)	% (95% CI)	TN/(TN + FN)	% (95% CI)
Bacteria (no. samples)												
*Escherichia coli* K1 (575)	100	2.5–100	100	99.4–100	50	1.3–98.7	100	99.4–100	100	N/A	99.8	99.3–100
*Haemophilus influenzae (575)*	N/A	N/A	99.8	99.0–100	N/A	N/A	99.8	99.0–100	0	N/A	100	N/A
*Listeria monocytogenes (575)*	100	2.5–100	99.7	98.7100	100	29.4–100	100	99.4–100	100	N/A	100	N/A
*Neisseria meningitidis (575)*	100	15.8–100	100	99.4–100	100	15.8–100	100	99.4–100	100	N/A	100	N/A
*Streptococcus agalactiae (575)*	100	47.8–100	99.3	98.2–99.8	100	66.1–100	99.8	99.0–100	88.9	53.0–98.3	100	N/A
*Streptococcus pneumoniae (575)*	100	47.8–100	99.8	99.0–100	100	47.8–100	99.8	99.0–100	83.3	41.4–97.3	100	N/A
Viruses (no. samples)												
Cytomegalovirus (27)	100	86.8–100	100	86.8–100	100	2.5–100	100	86.8–100	100	N/A	100	N/A
Enterovirus (52)	100	15.8–100	100	92.9–100	66,7	94.0–99.2	100	92.7–100	100	N/A	98	90.8–99.6
Herpes simplex virus 1 (105)	100	29.2–100	100	96.4–100	100	29.2–100	100	96.4–100	100	N/A	100	N/A
Herpes simplex virus 2 (105)	100	47.8–100	100	96.4–100	83,3	35.9–99.6	100	96.4–100	100	N/A	99	94.3–99.8
Human herpes virus 6 (13)	100	29.2–100	50	9.9–81.6	100	29.2–100	42.8	9.9–81.6	42.8	9.9–81.6	100	N/A
Human parechovirus (37)	N/A	N/A	100	90.5–100	N/A	N/A	100	90.5–100	N/A	N/A	100	N/A
Varicella-zoster virus (105)	100	79.4–100	94,4	87.4–98.2	100	83.2–100	98,8	93.6–100	95,2	74.0–99.3	100	N/A
Yeast (no. samples)												
*Cryptococcus neoformans/C. gattii (26)*	N/A	N/A	100	86.8–100	N/A	N/A	100	86.8–100	N/A	N/A	100	N/A

In the leftmost column, the number of samples tested with the respective comparator analysis or analyses, and thus included in the analyses in the table, is listed in parenthesis.

*TP* true positive, *FN* false negative, *TN* true negative, *FP* false positive.

When performance of FAME-p was compared against CDD, the PPA of FAME-p for *E. coli* K1, EV and HSV-2 was lower than when compared to microbiological comparator analyses (from 100 to 50%, from 100 to 66.7% and from 100 to 83.3%, respectively), though with overlapping confidence intervals (Table 3). Note that the number of patients and samples vary for different pathogens, as only samples tested with the respective CA for the pathogen in question were included in the analysis. In contrast, for *L. monocytogenes*, *S. agalactiae* and VZV the PPA was somewhat higher when the FAME-p results were compared against the clinical diagnosis at discharge than when compared to the microbiological comparator analyses (from 99.7 to 100%, from 99.3 to 99.8% and from 94.4 to 98.8%, respectively), although with overlapping confidence intervals. For HHV-6, however, the NPA of FAME-p was even lower when compared to the clinical diagnosis at discharged instead of the microbiological comparator analysis (from 50 to 42.8%), although the confidence intervals were overlapping. The PPV of FAME-p was 100% for seven analytes: *E. coli* K1, *L. monocytogenes, N. meningitidis*, CMV, EV, HSV-1, HSV-2, HPeV and *C. neoformans/C. gattii*. For *S. agalactiae, S. pneumoniae* and VZV, the PPV was lower (83.3–95.2). For *H. influenzae* and HHV-6, PPV was 0% and 42.8%, respectively. The NPV of FAME-p was 100% for all analytes except for *E. coli* (99.8%), EV (98%) and HSV-2 (99%). NPV calculation was not applicable for *C. neoformans/C. gattii* as no samples tested positive for *Cryptococcus* and no clinical diagnosis of cryptococcal infection was given.

### Discrepancy investigation

For the bacterial analytes, which used culture and PCR as the comparator, eight discrepantly positive results were considered: four for *S. agalactiae*, two for *L. monocytogenes*, one for *S. pneumoniae*, and one for *H. influenzae* (Table 4). Concerning *S. agalactiae*, three of four cases received diagnosis of *S. agalactiae* meningitis based on biochemical, clinical and radiological data. Positive FAME-p result was considered false positive in the remaining case who received CDD of hip fracture and fever. Although the two cases positive for *L. monocytogenes* in FAME-p were not confirmed by 16S rDNA PCR and culture, *L. monocytogenes* was found in blood cultures and both cases received a diagnosis of *L monocytogenes* meningitis. For the single cases of *S. pneumoniae* and *H. influenzae,* FAME-p results were considered false positives and patients received diagnoses of demyelinating disease and subarachnoid hemorrhage, respectively. In comparison to CDD, one discordantly negative result was considered for *E. coli*: despite negative FAME-p, culture and PCR, the patient received diagnosis of *E. coli* meningitis based on radiological findings and positive blood culture for *E. coli*.

**Table 4:** Results of discrepancy investigation for FAME-p based on comparator testing and clinical diagnosis at discharge.

Analyte	Discrepancy investigation
Enterovirus (n = 1)	Although both FAME-p and in-house PCR were negative, patient received diagnosis of EV meningitis based on biochemical analysis of CSF and detection of EV RNA in a specimen from throat
*Escherichia coli* (n = 1)	Although FAME-p, 16S rDNA PCR and CSF culture were negative, patient received diagnosis of *E. coli* meningitis based on positive blood culture for *E. coli* and radiological findings
*Haemophilus influenzae* (n = 1)	Positive FAME-p was not confirmed by in-house PCR and culture. Patient recieved clinical diagnosis of subarachnoidal hemorrhage
Herpes simplex virus 2 (n = 1)	Although both FAME-p and in-house PCR were negative, CNS infection with HSV-2 was confirmed radiologically and clinically
Human herpes virus 6 (n = 8)	Although both FAME-p and in-house PCR were positive, HHV-6 finding regarded as a bystander in three cases. In five cases, the positive FAME-p was discordant with in-house PCR and the patients did not receive a clinical diagnosis of HHV-6 infection
*Listeria monocytogenes* (n = 2)	Positive FAME-p was not confirmed by 16S rDNA PCR and culture. However, both cases recieved clinical diagnosis of *L. monocytogenes* meningitis based on positive blood culture for *L. monocytogenes*
*Streptococcus agalactiae* (n = 4)	Three cases received clinical diagnosis of meningitis, with *S. agalactiae* as the cause. One case was deemed to be a probable false positive, with the patient having a broken hip and fever, but no obvious meningitis
*Streptococcus pneumoniae* (n = 1)	Positive FAME-p was not confirmed by in-house PCR and culture. This case considered false positive and received a diagnosis of demyelinizing disease
Varicella-zoster virus (n = 5)	Positive FAME-p was not confirmed by in-house PCR. However, four cases received diagnosis of CNS infection secondary to varicerlla-zoster virus. One case considered false positive and received diagnosis of neuroborreliosis as a result of positive serology for *Borrelia* species

For the viral agents, which used in-house PCR as the comparator, 15 discordantly positive results were considered: eight for HHV-6 and five for VZV. HHV-6 positivity in those eight cases was considered clinically irrelevant. Four cases with positive FAME-p for VZV were not confirmed by in-house PCR. However, all four received clinical diagnoses of CNS infection secondary to VZV. In comparison to CDD, one discordantly negative result was considered for HSV-2: Although both FAME-p and in-house PCR were negative, CNS infection with HSV-2 was confirmed radiologically and clinically.

### Analysis of negative FAME-p

**Figure 1:**
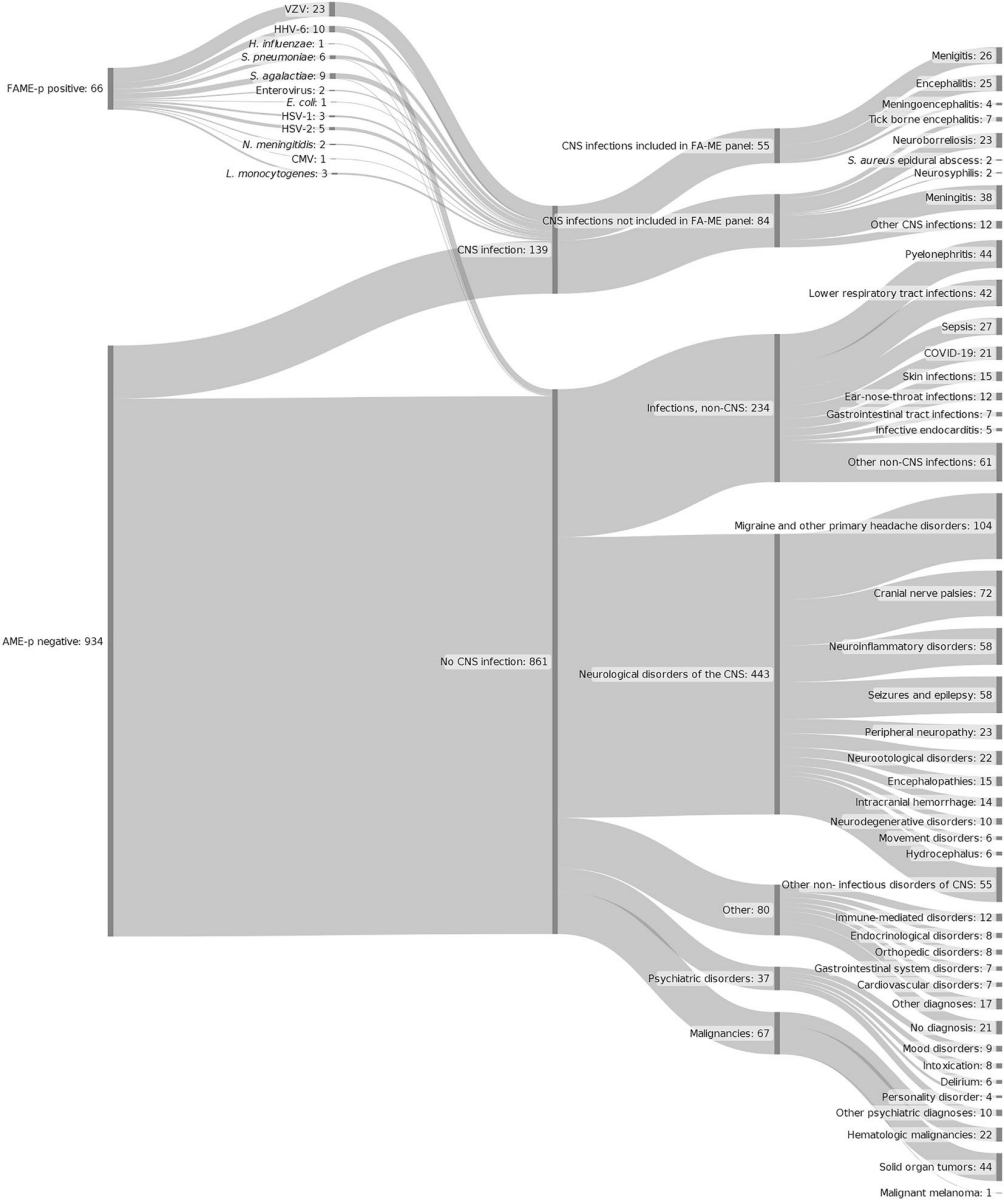
Plot of patients included in the study showing results in FAME-p analysis and final clinical diagnosis.

Among patients who were negative in FAME-p, 8.4% had a diagnosis of CNS infection. The most prevalent microorganisms in this group were *Borrelia* species (n = 23) and tick-borne encephalitis virus (TBE-virus, n = 7) (Fig. 1). Among patients who did not have a CNS infection as CDD, the five most common etiologies were migraine and other primary headache disorders (n = 104), cranial nerve palsies (n = 72), neuroinflammatory disorders (n = 58), seizure and epilepsy (n = 58) and pyelonephritis (n = 44).

### Other microbiological analyses

Apart from the comparator analyses, several other microbiological analyses of CSF were performed in some of the included patients. In 27 patients a specific fungal culture was performed on CSF (detecting one case of *Aspergillus* deemed to be a contaminant), and 37 patients were analyzed for SARS-CoV-2 in CSF using PCR (all negative). In 10 patients analyses for *Treponema pallidum* (TPPA and VDRL) were performed and was positive in one case. Analysis for neuroborreliosis (using CSF serology) was performed in 379 patients, of which 23 were diagnosed with neuroborreliosis. 143 patients underwent serological analysis for tick-borne encephalitis (TBE), of which 7 received a TBE diagnosis.

## Discussion

To our knowledge, this study represents the first observational study on contemporaneous comparison of the positive and negative results of FAME-p with microbiological assays and CDD. Review of the medical records for all FAME-p specimens enabled us, for the first time, to characterize the disease spectrum in patients referred for microbiological diagnosis of CNS infection (Figure 1), as well as to calculate PPV and NPV of individual agents in FAME-p which is one of the major strengths of this study.

CNS infection was diagnosed in only 13.9% of the patients who underwent FAME-p. Interestingly, in more than half of these patients, the identified agents were not included in FAME-p.

### FAME-p viral targets

FAME-p NPA compared to in-house VZV PCR was 94.4%, implying a risk of false positive result. However, four of five patients with negative in-house VZV PCR, had a diagnosis of VZV-encephalitis by radiological findings (Supplementary Table 1). One patient was diagnosed with neuroborreliosis, raising the question of cross-reactivity. Thereby, NPA of FAME-p for VZV compared to CDD increased to 98.8%. A positive FAME-p for VZV should be interpreted with caution to prevent overuse of antivirals and delay in correct diagnosis.

FAME-p PPA and NPV for HSV-1 compared to in-house PCR and CDD was calculated at 100%. However, based on previous data (5), adding in-house PCR in patients with high clinical suspicion, but negative FAME-p, is reasonable. Concerning HSV-2, we obtained 100% PPA and NPA of FAME-p compared to in-house PCR. However, one patient received diagnosis of HSV-2 meningitis despite negative FAME-p and in-house PCR. This was a patient with recurrent HSV-2 meningitis. Low viral load secondary to presence of intrathecal antibodies might be responsible for the discrepancy in this case. This issue has been previously described^15^.

Incidence of EV and HPeV in our study was lower compared to a recently published Swedish study by Lindström et al.^5^ which included 4199 samples obtained between 2017 and 2020 (EV: 2/1000 vs 109/4199 samples; HPeV: 0/1000 vs 7/4199 samples). Preventive SARS-CoV-2 measures might have role on reduced incidence of these viruses in our study. Although PPA of FAME-p for EV compared to in-house PCR was 100%, we observed a false-negative result in a patient who received diagnosis of EV meningitis based on detection of EV RNA in a specimen from throat (Supplementary Table 1). This resulted in decrease in PPA to 66.7% and NPV to 98%. It is well-known that enterovirus detection in CSF could be transient, and microbiological analysis of CSF can be complemented with PCR-analysis in specimens from throat and stool^16^. HHV-6 was identified in ten patients, however, it was regarded as a bystander or false in eight cases. Detection of HHV-6 in FAME-p may represent primary or latent infection and clinical relevance of HHV-6 detection in immunocompetent individuals is uncertain. Therefore, interpretation of HHV-6 positivity in FAME-p requires caution, and the utility of including HHV-6 in panels used for the diagnostic workup in community acquired meningitis and encephalitis has been the subject of debate^11^.

### FAME-p bacterial targets

FAME-p PPA for bacterial agents compared to culture and PCR was 100% (not applicable for *H. influenzae*). This implies a low risk of missing a CNS infection caused by any of the pathogens covered by FAME-p. However, NPA of FAME-p was calculated at 99.3–99.8% for *H. influenzae, L. monocytogenes, S. agalactiae* and *S. pneumoniae* (Table 3), implying a risk of false-positive results with the potential consequence of overuse of antibiotics and delay in correct diagnosis. Interestingly, discrepancy analysis compared to CDD revealed improvement in NPA of FAME-p for *L. monocytogenes* and *S. agalactiae*. However, NPA of FAME-p for *E. coli* compared to CDD resulted in a decline in NPA, due to a patient who received diagnosis of *E. coli* meningitis based on positive blood culture for *E. coli* (Supplementary Table 1). According to medical records, antibiotics were initiated 8 h before lumbar puncture, which may have affected the performance of the panel, as well as CSF culture, in this case.

This study has several limitations. Many pathogens possible to detect by FAME-p where not present, or only present in low numbers, which necessitates cautious interpretation of estimations of diagnostic performance. The fact that the comparator assays where not uniformly performed on all samples due to the retrospective study design represents another limitation. This is reflected in the large confidence intervals for pathogens where few samples were tested with the relevant comparator analysis, such as CMV. Another important limitation stemming from the retrospective design is that the results of FAME-p were available to the treating physicians determining the clinical diagnosis. This means that the results of FAME-p have influenced the clinical diagnosis which may lead to an over-estimation of the FAME-p correlation to clinical diagnosis. There is also a risk of negative results of both FAME-p and the microbiological comparator analyses despite an actual CNS infection with pathogens covered by the analyses. This could be due to limited sensitivity of the comparator assays as well or due to microbes dying or degrading during the time between sampling and the sample reaching the laboratory. It could also be due to very low concentrations of microbes in the CSF (such as is often the case with enteroviruses). The case of a clinically diagnosed HSV-2 CNS infection being negative in both FAME-p and the in-house PCR in CSF might also represent such an entity, as patients with a reactivation of HSV-2 often have antibodies limiting viral replication, and sometimes leading to low concentration in CSF^17^.

## Conclusions

In conclusion, although FAME-p shows good performance in routine microbiological diagnosis of CNS infections, result of FAME-p should be interpreted carefully together with results from other microbiological, radiological and biochemical tests. Pathogens not included in the panel should always be considered based on the local epidemiological situation. Also, since 86.1% of samples were from patients that were not diagnosed with CNS infection, we recommend including neurological and non-CNS infectious diseases in the differential diagnosis in patients presenting with symptoms compatible with CNS infection. We believe that continued studies on performance of FAME-p using two-sided discrepancy analysis are needed to increase the knowledge on clinical interpretation of FAME-p results, as well as optimal the optimal use of FAME-p in the clinic.

### Supplementary Information

Supplementary Table 1.
