# Supplementary material for: A retrospective observational study of 1000 consecutive patients tested with the FilmArray® Meningitis/Encephalitis panel: clinical diagnosis at discharge and microbiological findings
Source: Sci Rep. 2024 Feb 18;14:4015. doi: 10.1038/s41598-024-54621-9 (PMC10874959; doi:10.1038/s41598-024-54621-9)
Supplement: Supplementary file 1 — Supplementary Table 1. [file 41598_2024_54621_MOESM1_ESM.docx]

**Supplementary table.** Discrepancy analysis of all 66 FAME-p positive and 3 discordant FAME-p negative samples

*N/P: Not performed.

| **Age (year)** | **Gender** | **CSF analysis** | | | | | | | **FAME-p result** | **Respective comparator analysis and result** | **Clinical interpretation and notes of interest** | **Discrepancy analysis** |
| --- | --- | --- | --- | --- | --- | --- | --- | --- | --- | --- | --- | --- |
|  |  | **PMNL (cells/µl)** | **MNL (cells/µl)** | | **Protein (mg/L)** | **Albumin (mmol/L)** | **CSF/blood glucose ratio** | **Lactate (mmol/L)** |  |  |  |  |
| 77 | Female | 1004 | 193 | | N/P* | 1500 | 0.314 | 10,1 | CMV | In-house CMV PCR positive | *E. faecalis* meningitis and CMV reactivation. | True positive |
| 27 | Male | 27 | 0 | | 4 | 43 | N/P | N/P | Enterovirus | In-house enterovirus PCR positive | Enterovirus meningitis | True positive |
| 15 | Male | 15 | 0 | | 0 | 242 | N/P | 329 | Enterovirus | In-house enterovirus PCR positive | Enterovirus meningitis | True positive |
| 0 | Female | 404 | 138 | | N/P | 1413 | 0,3 | 4.8 | *E. coli* | *E. coli* in CSF culture, 16S rDNA PCR negative. | *E. coli* meningitis | True positive |
| 56 | Female | 4 | 14 | | 0.24 | 127 | 0,7 | 2.5 | *H. influenzae* | CSF culture and 16S rDNA PCR negative | Diagnosed with subarachnoidal hemorrhage. | False positive |
| 49 | Male | 0 | 38 | | 0.58 | 364 | 0.429 | 1.5 | HSV-1 | In-house HSV-1 PCR positive | HSV-1 encephalitis | True positive |
| 66 | Female | 0 | 53 | | N/P | N/P | 0.74 | 3.3 | HSV-1 | In-house HSV-1 PCR positive | HSV-1 encephalitis | True positive |
| 5 | Male | 5 | 0 | | 5 | 246 | 0.37 | 173 | HSV-1 | In-house HSV-1 PCR positive | HSV-1 encephalitis | True positive |
| 32 | Female | 13 | 387 | | N/P | N/P | N/P | N/P | HSV-2 | In-house HSV-2 PCR positive | HSV-2 meningitis | True positive |
| 32 | Female | 29 | 1117 | | N/P | N/P | N/P | 3.9 | HSV-2 | In-house HSV-2 PCR positive | HSV-2 meningitis | True positive |
| 63 | Female | 63 | 1 | | 7 | 169 | N/P | 357 | HSV-2 | In-house HSV-2 PCR positive | HSV-2 meningitis | True positive |
| 50 | Female | 50 | 1 | | 0 | 163 | 1 | 700 | HSV-2 | In-house HSV-2 PCR positive | HSV-2 meningitis | True positive |
| 65 | Female | 65 | 1 | | 0 | 224 | N/P | 438 | HSV-2 | In-house HSV-2 PCR positive | HSV-2 meningitis | True positive |
| 11 | Female | 0 | 305 | | 0.76 | 505 | 0.6 | 2.6 | HHV-6 | External in-house HHV-6 PCR positive | Diagnosed with HaNDL syndrome, HHV-6 interpreted as a true finding but not clinically relevant. | True positive |
| 31 | Female | 0 | 0 | | 0.37 | 245 | 0.6 | 1.6 | HHV-6 | External in-house HHV-6 PCR positive | Diagnosed with fever of uncertain cause, HHV-6 interpreted as a bystander. | True positive |
| 33 | Male | 0 | 305 | | N/P | 905 | 0.6 | 1.9 | HHV-6 | External in-house HHV-6 PCR negative | Diagnosed with HaNDL syndrome. | False positive |
| 38 | Female | 0 | 3 | | N/P | 286 | 0.782 | 2.1 | HHV-6 | External in-house HHV-6 PCR positive | HHV-6 meningitis | True positive |
| 2 | Female | 0 | 0 | | N/P | 85 | N/P | 1.7 | HHV-6 | External in-house HHV-6 PCR positive | HHV-6 meningitis with fever cramps | True positive |
| 80 | Male | 80 | 0 | | 0 | 0 | N/P | 254 | HHV-6 | External in-house HHV-6 PCR negative | Confusion of unknown cause | False positive |
| 0 | Male | 0 | 0 | | 0 | 4 | 0,61 | 363 | HHV-6 | External in-house HHV-6 PCR negative | Pyelonephritis | False positive |
| 32 | Male | 32 | 0 | | 0 | 7 | 0.33 | 234 | HHV-6 | External in-house HHV-6 PCR negative | Multiple sclerosis | False positive |
| 37 | Female | 37 | 1 | | 0 | 0 | 0.27 | 164 | HHV-6 | External in-house HHV-6 PCR positive | Diagnosed with pneumonia, HHV-6 interpreted as a true finding but not clinically relevant. | True positive |
| 61 | Female | 0 | 0 | | 0.42 | 246 | 0.7 | 1.9 | HHV-6 | External in-house HHV-6 PCR negative | COVID-19 | False positive |
| 62 | Female | 27 | 166 | | N/P | 1281 | 0.147 | 14.1 | *L. monocytogenes* | CSF culture positive | *L. monocytogenes* meningitis | True positive |
| 88 | Female | 88 | 1 | | 5 | 0 | N/P | 1130 | *L. monocytogenes* | CSF culture negative, 16s rDNA PCR negative. Blood culture positive for *L. monocytogenes*. | *L. monocytogenes* meningitis, diagnosis based on clinical picture and positive blood culture. | True positive |
| 85 | Male | 85 | 0 | | 0 | 189 | N/P | 2561 | *L. monocytogenes* | CSF culture negative, 16s rDNA PCR negative. Blood culture positive for *L. monocytogenes*. | *L. monocytogenes* meningitis, diagnosis based on clinical picture and positive blood culture. | True positive |
| 0 | Male | 0 | 32 | | N/P | N/P | 1 | 7.6 | *N. meningitidis* | CSF culture negative. In-house PCR positive for *N. meningitidis.* | *N. meningitidis* meningitis with sepsis | True positive |
| 37 | Male | 22581 | 420 | | N/P | 3235 | 0.185 | 17 | *N. meningitidis* | CSF culture negative. Blood culture positive for *N. meningitidis*. In house PCR positive for *N. meningitidis*. | *N. meningitidis* meningitis | True positive |
| 75 | Male | 3647 | 496 | | 4.5 | 2720 | 0.3 | 9.5 | *S. agalactiae* | CSF culture and 16S rDNA PCR analysis negative. | Meningitis with *S. agalactiae* based on clinical and radiological interpretation. | True positive |
| 0 | Male | N/P | N/P | | N/P | N/P | N/P | N/P | *S. agalactiae* | CSF culture and 16S rDNA PCR analysis negative. CSF culture one week later was positive. Throat swap was positive for *S. agalactiae*. | *S. agalactiae* meningitis | True positive |
| 0 | Female | 144 | 197 | | 4.6 | 2893 | 0,02 | 11.2 | *S. agalactiae* | CSF culture negative. | *S. agalactiae* meningitis based on clinical and radiological interpretation. | True positive |
| 0 | Female | 1054 | 474 | | N/P | 3392 | 0,02 | 9.2 | *S. agalactiae* | CSF culture and 16S rDNA PCR positive | *S. agalactiae* meningitis | True positive |
| 0 | Male | 9391 | 1001 | | 2.4 | 1495 | 0,4 | 8.8 | *S. agalactiae* | CSF culture and 16S rDNA PCR positive | *S. agalactiae* meningitis | True positive |
| 0 | Female | 432 | 13 | | N/P | 600 | 0.5 | 4.3 | *S. agalactiae* | CSF culture and 16S rDNA PCR positive | *S. agalactiae* meningitis | True positive |
| 0 | Female | 38 | 31 | | N/P | N/P | 0,032 | 8.6 | *S. agalactiae* | CSF culture and 16S rDNA PCR positive | *S. agalactiae* meningitis | True positive |
| 86 | Male | 0 | 0 | | N/P | 307 | 0,506 | 2.4 | *S. agalactiae* | CSF culture and 16s rDNA PCR negative | Hip fracture | False positive |
| 0 | Male | 0 | 0 | | 308 | 277 | 0.85 | 556 | *S. agalactiae* | CSF culture positive | *S. agalactiae* meningitis | True positive |
| 65 | Male | 4124 | 783 | | N/P | 1744 | 0.02 | 15 | *S. pneumoniae* | CSF culture and in-house *S. pneumoniae* PCR positive*.* | *S. pneumoniae* meningitis | True positive |
| 8 | Male | 9999 | 1320 | | 2.9 | 1920 | 0,5 | 6.4 | *S. pneumoniae* | CSF culture negative, in-house *S. pneumoniae* PCR positive. | *S. pneumoniae* meningitis | True positive |
| 66 | Female | 1677 | 17 | | 2.5 | 1739 | 0.106 | 10.3 | *S. pneumoniae* | CSF culture and in-house *S. pneumoniae* PCR positive. | *S. pneumoniae* meningitis | True positive |
| 13 | Female | 0 | 4 | | N/P | 143 | N/P | 2.2 | *S. pneumoniae* | CSF culture, PCR (in-house and 16S rDNA) negative | Demyelinizing disease | False positive |
| 14 | Female | 11234 | 38 | | N/P | 1824 | 0.238 | 8.7 | *S. pneumoniae* | CSF culture, microscopy and in-house *S. pneumoniae* PCR positive. | *S. pneumoniae* meningitis | True positive |
| 65 | Male | 65 | 0 | | 147 | 378 | N/P | 1266 | *S. pneumoniae* | CSF culture negative. Blood culture positive for *S. pneumoniae*. | *S. pneumoniae* meningitis based on clinical picture and blood culture. | True positive |
| 57 | Male | 0 | 185 | | 0.75 | 460 | 0.7 | 1.9 | VZV | In-house VZV PCR positive | VZV meningitis | True positive |
| 33 | Female | 0 | 126 | | 1 | 659 | 0,6 | 2.6 | VZV | In-house VZV PCR positive | VZV meningitis | True positive |
| 3 | Male | 0 | 4 | | 0.16 | 77 | N/P | 1.2 | VZV | In-house VZV PCR positive | VZV meningitis | True positive |
| 72 | Female | 0 | 572 | | 1 | 704 | N/P | 2.6 | VZV | In-house VZV PCR positive | VZV meningitis | True positive |
| 34 | Male | 0 | 601 | | N/P | 1873 | 0.6 | 3.3 | VZV | In-house VZV PCR positive | VZV meningitis | True positive |
| 44 | Male | 4 | 299 | | N/P | 900 | 0.4 | 3.4 | VZV | In-house VZV PCR positive | VZV meningitis | True positive |
| 57 | Male | 0 | 57 | | 0.72 | 441 | 0.5 | 2,9 | VZV | In-house VZV PCR positive | VZV meningitis | True positive |
| 4 | Female | 6 | 191 | | 0.74 | 466 | 0.6 | 1.5 | VZV | In-house VZV PCR negative, CSF culture negative, CSF serology positive for *Borrelia* species. | Neuroborreliosis | False positive |
| 69 | Male | 0 | 26 | | 1 | 620 | 0.596 | 3.4 | VZV | In-house VZV PCR negative | VZV meningitis | True positive |
| 75 | Female | 0 | 168 | | 0.76 | 463 | N/P | 2.7 | VZV | In-house VZV PCR positive | VZV encephalitis | True positive |
| 35 | Female | 0 | 58 | | N/P | 238 | 0.547 | 2.6 | VZV | In-house VZV PCR negative | VZV meningitis | True positive |
| 78 | Female | 4 | 133 | | N/P | 404 | 0.365 | 2.8 | VZV | In-house VZV PCR positive | VZV meningitis | True positive |
| 75 | Female | 0 | 10 | | N/P | 1026 | 0.515 | 3.1 | VZV | In-house VZV PCR positive | VZV meningitis with shingles | True positive |
| 78 | Female | 0 | 19 | | N/P | N/P | 0.38 | 3.1 | VZV | In-house VZV PCR negative | VZV meningitis | True positive |
| 72 | Male | 0 | 55 | | 0,9 | 549 | 0.522 | 1.5 | VZV | In-house VZV PCR positive | VZV encephalitis | True positive |
| 53 | Female | 0 | 4 | | N/P | 158 | 0.7 | 1.5 | VZV | In-house VZV PCR positive | VZV meningitis with zoster ophtalmicus | True positive |
| 76 | Female | 1 | 16 | | 41 | 2.6 | 1673 | 0.4 | VZV | In-house VZV PCR positive | VZV encephalitis | True positive |
| 55 | Female | 55 | 1 | | 0 | 0 | 0.3 | 202 | VZV | In-house VZV PCR positive in CSF and fluid from blister. | VZV meningitis | True positive |
| 21 | Female | 21 | 1 | | 3 | 1438 | N/P | N/P | VZV | In-house VZV PCR positive | VZV meningitis | True positive |
| 42 | Female | 42 | 1 | | 0 | 271 | N/P | 374 | VZV | In-house VZV PCR positive | VZV meningitis | True positive |
| 15 | Male | 15 | 0 | | 0 | 0 | N/P | 140 | VZV | In-house VZV PCR negative | VZV encephalitis | True positive |
| 21 | Female | 21 | 1 | | 0 | 552 | 0,72 | 502 | VZV | In-house VZV PCR positive | VZV meningitis | True positive |
| 23 | Female | 23 | 1 | | 0 | 435 | 0,75 | 531 | VZV | In-house VZV PCR positive | VZV meningitis | True positive |
| 40 | Male | 0 | 152 | | N/P | 297 | N/P | 2.5 | Negative | In-house PCR negative | Recurrent HSV-2 meningitis, based on radiological and clinical picture and previous history of HSV-2 meningitis. | False negative |
| 0 | Female | 0 | 44 | | 0.22 | 130 | N/P | 1.5 | Negative | In-house enterovirus PCR negative in CSF but positive in sample from throat. | Enterovirus meningitis, diagnosis based on clinical picture, biochemical analyses and enterovirus RNA i sample from throat. | False negative |
| 80 | Female | 4540 | 339 | | N/P | 5638 | N/P | N/P | Negative | Culture and 16S rDNA PCR from CSF were negative. *E. coli* in blood culture. | Diagnosis of *E. coli* meningitis based on blood culture, clinical and radiological findings. | False negative |
|  | | | |  |  |  |  |  |  |  |  |  |
